# A longitudinal study on quality of life after injury in children

**DOI:** 10.1186/s12955-016-0523-6

**Published:** 2016-08-26

**Authors:** Amy Schneeberg, Takuro Ishikawa, Sami Kruse, Erica Zallen, Craig Mitton, Julie A. Bettinger, Mariana Brussoni

**Affiliations:** 1 School of Population & Public Health, University of British Columbia, Vancouver, BC Canada; 2 British Columbia Injury Research & Prevention Unit, F508 – 4480 Oak Street, Vancouver, BC V6H 3 V4 Canada; 3 British Columbia Children’s Hospital, Vancouver, BC Canada; 4 Department of Pediatrics, University of British Columbia, Vancouver, BC Canada; 5 Vaccine Evaluation Center, BC Children’s Hospital, Vancouver, Canada

**Keywords:** Pediatrics, Unintentional injury, Health related quality of life

## Abstract

**Background:**

In high income countries, injuries account for 40 % of all child deaths, representing the leading cause of child mortality and a major source of morbidity. The need for studies across age groups, and use of health related quality of life measures that assess functional limitations in multiple health domains, with sampling at specific post-injury time points has been identified. The objective of this study was to describe the impact of childhood injury and recovery on health related quality of life (HRQoL) for the 12 months after injury.

**Methods:**

In this prospective cohort study parents of children 0-16 years old attending British Columbia Children’s Hospital for an injury were surveyed over 12 months post-injury. Surveys assessed HRQoL at four points: baseline (pre-injury), one month, four to six months and 12 months post injury. Generalized estimating equation models identified factors associated with changes in HRQoL over time.

**Results:**

A total of 256 baseline surveys were completed. Response rates for follow-ups at one, four and twelve months were 74 % (186), 67 % (169) and 64 % (161), respectively. The mean age of participants was 7.9 years and 30 % were admitted to the hospital. At baseline, a retrospective measure of pre-injury health, the mean HRQoL score was 90.7. Mean HRQoL ratings at one, four and 12 months post injury were 77.8, 90.3 and 91.3, respectively. Both being older and being hospitalized were associated with a steeper slope to recovery.

**Conclusions:**

Although injuries are prevalent, the long term impacts of most childhood injuries are limited. Regardless of injury severity, most injured children recuperated quickly, and had regained total baseline status by four month post-injury. However, although hospitalization did not appear to impact long term psychosocial recovery, at four and 12 months post injury a greater proportion of hospitalized children continued to have depressed physical HRQoL scores. Both older and hospitalized children reported greater impact to HRQoL at one month post injury, and both had a steeper slope to recovery and were on par with their peers by four month.

## Background

Injuries account for 40 % of all child deaths in high-income countries, representing the leading cause of child mortality and a major source of morbidity [1]. In the United States, each year more than 9,000 children aged 19 and under die from unintentional injuries, and another 225,000 are hospitalized [2]. Although indicative of the enormous public health problem that injuries comprise, mortality and hospitalization statistics are inadequate to fully understand the burden of injury.

The World Health Organization’s definition of health includes physical, mental and social dimensions [3]. While physiologic measures of injury severity are important to clinicians, measures of functional capacity and wellbeing can be of greater importance to individuals [4]. Measuring health related quality of life (HRQoL) after injury facilitates quantifying the impact on multiple dimensions of health and the recovery process. As a multidimensional, patient centered outcome, HRQoL measures encompass a wide range of experiences to given health states, including physical and psychosocial function as well as spiritual wellbeing [5]. It is important to identify potential long-term impact of injuries on HRQoL in order to provide timely and ongoing support. Research following child injury on psychological outcomes, is currently investigating predictors and early intervention strategies, and the present study can help build towards these interventions [6–8].

Studies examining HRQoL after injuries in adult populations have found that the impacts of injury can vary in time and extent, and that injury severity is not the only predictive factor [9]. These studies typically focus on patients with severe injuries, limiting our understanding of outcomes for the majority of injuries which do not result in death or severe disability [10]. It has been estimated that for every individual who dies from an unintentional injury, there are approximately six other individuals who are hospitalized, 45 individuals with emergency department (ED) visits and 48 individuals with visits to primary healthcare providers [11]. Thus, it is important to consider the impact less severe injuries may have given the health care burden they represent.

Study methods used to assess post injury HRQoL vary widely with respect to sample population, measurement instruments used, and timing of measurements, and they rarely include a baseline HRQoL measurement for comparison to pre-injury state [10]. Further, studies with pediatric samples are rare despite the fact that the impact of childhood injuries can differ substantially from adults [9, 10]. Therefore, studies across age groups, and use of HRQoL measures that assess functional limitations in multiple health domains, with sampling at specific post-injury time points are needed [10]. Pediatric studies using generic measures of HRQoL with baseline measurements of health and wellbeing are required to understand the impact injuries have on this unique population. Available studies have found that the impact of injuries on HRQoL can extend well into the year following treatment. One study that examined the health status of children in the six months following admission to hospital for injury found that general health perceptions, physical functioning, social and physical roles, behavior, parental impact including emotional and family activities all remained lower at discharge, one month, and six months post-injury relative to a population of healthy children [12]. Although this study demonstrated that the impact of injuries can remain at six months post hospital admission for both the child and parents, they did not explore less severe injuries not requiring admission to hospital, nor did they explore factors, outside of severity, that may be used to predict depressed physical and emotional functioning. Other studies that included less severe injuries not requiring hospitalization, have found that a small proportion (8 %) of children still reported functional limitations at nine months post injury and that some injured children had depressed HRQoL scores up to two years post injury [13, 14], however these studies excluded very young children (< 5 years of age).

There are gaps in the literature regarding the immediate and long-term impacts of injury and recovery, particularly in pediatric populations. The objective of this research is to better understand the impact of a broad spectrum of childhood injuries of varying severity on HRQoL and to identify demographic and diagnostic variables associated with a significant relationship with HRQoL.

## Methods

### Study population

This study collected data longitudinally from a sample of parents of children aged 0 to 16 years who presented with a primary injury diagnosis at the British Columbia Children’s Hospital (BCCH) ED or were admitted to the hospital wards between February 2011 and December 2013. For children 0 to 5 years old, only parents completed surveys. For all other ages, both children and parents completed surveys. For consistency, the present paper presents only parents’ reports for all participants.

### Data collection

A research assistant recruited directly from the ED and hospital wards on different days of the week and times of day. In addition, real time hospital admissions data were reviewed twice daily during regular office hours to identify children presenting with injury for study recruitment. Because most medically attended visits for injuries do not result in hospitalization, injuries requiring hospitalization were proportionately over-sampled to ensure a mix of patients with injuries of varying severity. Thirty percent of the study sample was hospitalized relative to only 10 % of the population of all children presenting to the hospital with an injury.

**Fig. 1:**
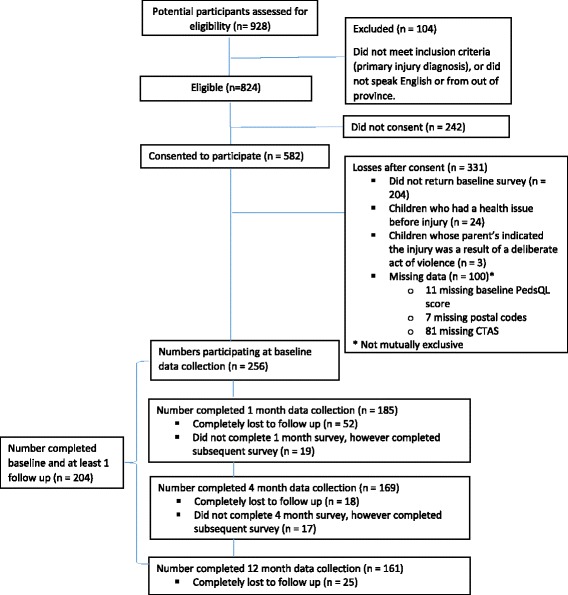
Study population disposition

Before approaching parents in hospital wards, researchers gained permission from the nurse or physician responsible for the child’s care. In the ED, parents were approached in waiting rooms, after triage confirmed that the primary reason for the visit was injury. All participants gave written consent. Parents who did not speak English or did not have an address in British Columbia (BC) were excluded from the sample. Twenty four parents indicated their child suffered from a disability or long-term health problem before the injury. Since these children had relatively rare conditions that can increase risk of injury and hospitalization their parents’ data were excluded from analysis. While we did not begin the study deliberately excluding intentional injuries (e.g., self-harm, assaults), we recognized that there would be different impacts on HRQoL. Only three participants with intentional injuries agreed to participate, which was insufficient for meaningful analysis, thus, they were excluded from the analyses reported herein. See Fig. 1 for a flowchart outlining participant disposition. This study was reviewed and approved by the University of British Columbia/Children’s and Women’s Health Centre of British Columbia Research Ethics Board.

A study specific survey instrument incorporating the Pediatric Quality of Life Questionnaire (PedsQL™) was administered to parents/guardians at baseline and one, four and 12 months post injury, as per guidelines outlined by van Beeck et al. [10]. At baseline, PedsQL™ was assessed as a retrospective measure of pre-injury health and asked about the child’s HRQoL in the month prior to injury. The questionnaire was piloted among a sample of 10 parents to ensure clarity and comprehension. Components of the questionnaire had previously been validated (the PedsQL [15, 16]) however, validity and reliability were not measured for questions related to demographics and the circumstances of the injury. Parents could complete a hard copy of the questionnaire and return in a stamped self-addressed envelope, or through an online link. Research shows, mode of administration (pen and paper, online or telephone) does not influence scores [17]. At the time of recruitment and with each subsequent follow-up, parents were offered a $2 gift card to a local coffee and pastries merchant for participating in the study, irrespective of whether they completed the survey or not.

### Descriptive variables

At baseline, the survey instrument included questions about the circumstances around the injury and demographic information including the child’s age, and sex. Hospital records were used to determine each child’s length of hospitalization Pediatric Canadian Triage and Acuity Scale (PaedCTAS) score. The PaedCTAS is a scale used to triage patients based on urgency. PaedCTAS has five ordinal categories ranging from 1 (requires resuscitation) to 5 (non-urgent), and it is assigned to all children presenting in Canadian EDs [18]. This score can be used to predict the nature and scope of care that is likely to be required. The scoring is highly standardized, as nurses assigning the score receive continuous training. Both PaedCTAS score and hospitalization status were used as independent proxies of injury severity. Research indicates the utility of the PaedCTAS as an alternate proxy of injury severity that is not as sensitive to extraneous factors that can influence hospitalization status [19]. Participants’ postal codes were used to derive a measure of socioeconomic status (neighbourhood income quintiles) using Statistics Canada’s Postal Code Conversion File Plus [20].

### Health related quality of life

The PedsQL™ 4.0 Generic Core and the PedsQL™ Infant Scales were developed to assess HRQoL in children, ages 2 to 18 years and 0 to 24 months, respectively. The PedsQL™ 4.0 Generic Core is a 23 item scale and includes four subscales: physical functioning, emotional functioning, social functioning and school functioning [21]. The PedsQL™ Infant Scale is an instrument composed of 45 items and five subscales: physical functioning, physical symptoms, emotional functioning, social functioning and cognitive functioning [22]. Both PedsQL™ instruments use a five point Likert response scale ranging from “never” to “almost always” to assess the extent to which different items have affected the child in the previous month. For both measures, individual item scores have been obtained by reverse scoring items and linearly transforming them to a scale of 0 to 100, with 100 representing perfect health. Total scores have been obtained by adding the sum of items and dividing them by the number of items answered. Studies that have reviewed tools for the purpose of long-term follow-up and assessing outcomes in pediatric trauma populations have identified the PedsQL™ as one of very few that is appropriate for a large age range that also has robust psychometric properties [22, 23]. A difference of 4.5 for parent proxy have been previously established as the minimal clinically meaningful difference for this tool [24]. To better understand the true burden of injury overtime both the mean HRQoL score at each time point, as well as the proportion of children who continue to report depressed HRQoL scores at each time point (prevalence of outstanding impact) has been investigated.

### Statistical analysis

Logistic regression was used to compare the final analytic sample, the children for whom at least one follow-up survey was available (*n* = 204), to the entire sample of children presenting with injury to BCCH wards or ED during the study period using administrative data obtained from BCCH. BCCH administrative data included postal code, sex, age, length of stay and hospitalization status. Statistics Canada’s Postal Code Conversion File Plus was used to assign neighbourhood income quintiles.

The HRQoL of study participants at baseline and follow-up points was measured using parent response to PedsQL™. The relationship between PedsQL™ score at each time point and demographic and injury related variables was explored using bivariable linear regression. To determine the proportion of children who continued to have depressed HRQoL scores relative to baseline, the “prevalence of outstanding impact”, on overall HRQoL as well as the physical and psychosocial domains independently at each time point, was defined as having a score that was at least 1 standard deviation of the baseline mean below the individual’s baseline score. The relationship between the prevalence of outstanding impact on HRQoL and injury severity was explored by investigating the relationship between “prevalence of impaired HRQoL” and hospitalization status and PaedCTAS scores using chi-square or Fisher’s exact tests, as appropriate. A Bonferroni correction was applied to determine the alpha used for statistical significance to account for multiple comparisons.

Bivariable associations between demographic and injury related variables were investigated with chi square tests for categorical comparisons and t-tests or ANOVA for continuous (age) variables. If variables were identified to be potentially collinear (*p* < 0.10) the variable with a stronger crude relationship with recovery in HRQoL over time was brought forward for model building. Bivariable generalized estimating equation (GEE) models using an exchangeable covariance matrix were built to explore the crude impact of demographic and injury related variables on HRQoL. To explore the impact of independent variables on recovery overtime an interaction term with time was included in all models. For the purpose of model building PaedCTAS scores were collapsed into 3 categories, PaedCTAS 1 and 2, 3 and 4 and 5, because there were not enough cases in the highest and lowest categories. A multivariable GEE model was built including all variables identified to be statistically or conceptually important. The model was run with all observations in the analytic sample (*n* = 204). Finally, a sensitivity analysis was run to assess the impact of exclusions due to missing data on our results. The mean HRQoL scores for the analytic sample at each time point were compared to the mean scores of the entire population of children who returned any data over the study period and no clinically or statistically significant differences were identified (results not shown).

## Results

### Study population

After exclusions there were 256 baseline surveys; of those individuals 204 returned at least one follow-up survey, making up the analytic sample. Table 1 provides demographic and injury information for study participants included in the analytic sample and a comparison of participants with complete data to those lost to follow-up. The analytic sample was not statistically significantly different than the sample of children who returned a baseline survey on any of the demographic of injury related variables collected (results not shown). Also, the analytic sample was not statistically different than the broader population of all injured children based on sex or age, however the children in our sample had higher odds of being hospitalized (a result of purposeful sampling) and lower odds of being in the lowest 2 income quintiles relative to all injured children presenting at the hospital during the study period (Table 2).

**Table 1:** Baseline characteristics

		Attrition		
	Total *n* = 204	12 Month Complete *n* = 149	Lost to Follow-Up *n* = 55	OR^a^ (95 % CI)
Baseline HRQoL				
Mean (SD)	90.7 (±8.9)	91.3 (±8.3)	89.0 (±10.3)	1.0 (0.9, 1.1)
Hospitalization Status n (%)				
Emergency Department	144 (70.6)	108 (72.5)	35 (66.0)	0.7 (0.4, 1.4)
Hospitalized	60 (29.4)	41 (27.5)	20 (34.0)	
Length of Stay (days)				
Median (25 %, 75 %)	2.7 (1.5, 6.8)	2.5 (1.5, 5.0)	3.8 (1.8, 10.6)	0.9 (0.8, 1.0)
Range	0.2 – 43.4	0.2 – 14.9	0.2 – 43.4	
Sex n (%)				
Male	127 (62.3)	96 (64.4)	31 (56.4)	1.4 (0.7, 2.6)
Female	77 (37.7)	53 (35.6)	24 (43.6)	Ref
Age (years)				
Median (25 %, 75 %)	7.1 (3.6, 11.7)	7.3 (3.7, 11.9)	6.8 (3.0, 11.6)	1.0 (0.9, 1.1)
Range	0.1 - 16.9	0.3 – 16.9	0.1 – 16.6	
Age Category n (%)				
0 – 5 years	84 (41.2)	62 (41.6)	22 (40.0)	0.9 (0.4, 2.0)
6 – 10 year	66 (32.4)	46 (30.9)	20 (36.4)	0.7 (0.3, 1.6)
11 -16 years	54 (26.5)	41 (27.5)	13 (23.6)	Ref
PaedCTAS n (%)				
1 (requires resuscitation)	11 (5.4)	8 (5.4)	3 (5.5)	0.4 (0.0, 4.6)
2	38 (18.6)	28 (18.8)	10 (18.2)	0.4 (0.0, 3.7)
3	43 (21.1)	30 (20.1)	13 (23.6)	0.3 (0.0, 3.0)
4	104 (51.0)	76 (51.0)	28 (50.9)	0.4 (0.0, 3.3)
5 (non-urgent)	8 (3.9)	7 (4.7)	1 (1.8)	Ref
Income Quintile n (%)				
1 (lowest income quintile)	25 (12.3)	12 (8.1)	13 (23.6)	0.3 (0.1, 0.7)
2	25 (12.3)	16 (10.7)	9 (16.4)	0.5 (0.2, 1.4)
3	43 (21.1)	37 (24.8)	6 (10.9)	1.8 (0.6, 4.9)
4	39 (19.1)	28 (18.8)	11 (20.0)	0.7 (0.3, 1.8)
5 (highest income quintile)	72 (35.3)	56 (37.6)	16 (29.1)	Ref
Injury Type				
Head injury	18 (8.8)	12 (8.1)	6 (11.3)	1.2 (0.3, 4.9)
Lower extremity fracture	25 (12.3)	15 (10.1)	10 (18.9)	0.9 (0.3, 3.3)
Major trauma	16 (7.8)	13 (8.8)	3 (5.7)	2.6 (0.5, 13.0)
Minor external injury	77 (37.7)	57 (38.5)	20 (37.7)	1.7 (0.6, 5.3)
Upper extremity fracture	49 (24.0)	41 (27.7)	8 (15.1)	3.1 (0.9, 10.9)
Other^b^	18 (9)	10 (6.8)	6 (11.3)	Ref
Missing	1 (0.5)	1 (1.0)	2 (2.3)	

^a^OR from logistic regression, odds of returning 12 month survey

^b^Categories with cell size < 5 were collapsed into, other this category includes Major Burn, Hand or foot amputation, Head Trauma, Ingestion/chocking, Internal organ injury, Spinal fracture

**Table 2:** Study population compared to all children presenting to hospital with injury during study period

	Study population	All injuries	OR (95 % CI)
Sex n (%)			
Male	127 (62.3)	8156 (58.3)	ref
Female	77 (37.8)	5825 (41.7)	1.2 (0.9, 1.6)
Income Quintile n (%)			
1(lowest income quintile)	25 (12.3)	2,744 (19.8)	0.4 (0.3, 0.7)
2	25 (12.3)	2,598 (18.8)	0.4 (0.3, 0.7)
3	43 (21.1)	2,607 (18.9)	0.8 (0.5, 1.1)
4	39 (19.1)	2,586 (18.9)	0.7 (0.5, 1.0)
5(hightest income quintile)	72 (35.3)	3,305 (23.9)	ref
Hospitalized n (%)			
ED	144 (70.6)	12,419 (88.8)	ref
Admitted	60 (29.4)	1,562 (11.2)	3.3 (2.4, 4.5)
Age (Mean ± SD) (range 0 – < 17 years)	7.87 ± 4.67	7.32 ± 5.02	1.0 (1.0. 1.1)

The mean age of analytic sample was 7.9 years, 62.3 % were male, and 89 % of respondents indicated English was the primary, or one of the primary languages spoken at home. Almost 30 % of participants were hospitalized for their injuries. The median length of stay for children who were hospitalized was 2.7 days, ranging from < 1 day to 43 days, with 40 % being hospitalized for 2 days or less. The majority of children included in this study were previously healthy with 87 % of parents indicating that their child had zero days of ill health in the four weeks preceding the injury. Most parents indicated their child was participating in leisure/entertainment activities (32 %), or sports/exercise either at school or at a club/gym (31 %) at the time of injury.

Table 3 presents HRQoL for participants based on PedsQL™ summary scores as reported by parents at each time point stratified by demographic and injury-related variables. The mean baseline total HRQoL score (representing pre-injury health) of participants was 90.66 (95 % CI (89.4, 91.9)). At one month the mean total health score dropped to 77.8 (95 % CI (75.2, 80.4)), by four months this mean returned to almost that of pre injury status (90.3 (88.9, 91.8)) and by 12 months the mean score was equal to pre injury status (91.3 (89.8, 92.8)). None of the demographic or injury related variables were statistically significantly associated with baseline or twelve month HRQoL summary scores. At one-month post injury having been hospitalized, having a lower PaedCTAS score and being over the age of 8 were all significantly associated with lower HRQoL summary scores (*p* < 0.001 due to Bonferroni correction). These relationships were no longer evident at four months post injury, except for age.

**Table 3:** PedsQL total health score, parent report at each follow-up point^a^,^*^

	Baseline			One Month			Four Months			Twelve Months		
	n	mean	sd	n	mean	sd	n	mean	sd	n	mean	sd
Sex		*p* = 0.31			*p* = 0.82			*p* = 0.47			*p* = 0.44	
Male	127	90.2	9.2	113	77.6	17.5	106	89.9	9.7	103	90.8	9.8
Female	77	91.5	8.3	72	78.2	18.6	63	91	9.6	58	92.1	9.5
Age Category		*p* = 0.58			***p*** ** = 0.005**			***p*** ** = 0.003**			*p* = 0.22	
0 - 5	84	91.3	8.5	74	82.8	16.0	65	92.4	7.9	62	92.2	9.6
6 -10	66	89.8	9.5	63	73.0	18.7	52	86.6	11.4	46	89.6	10.7
11-16	54	90.7	8.8	48	76.5	17.9	41	91.3	8.5	41	92.9	7.2
Hospitalization Status		*p* = 0.77			***p*** ** < 0.001**			*p* = 0.71			*p* = 0.82	
ED	144	90.5	8.9	132	82.6	14.8	126	90.2	10.1	113	91.4	9.2
Hospitalized	60	90.9	8.9	53	65.9	19.6	43	90.8	8.4	48	91	10.8
PaedCTAS		*p* = 0.15			***p*** ** < 0.001**			*p* = 0.72			*p* = 0.87	
1 (resuscitation required)	11	85.4	12.4	10	65.8	17	9	88	9.4	7	90.7	12
2	38	91.1	8.6	33	68.2	19.9	30	90.1	8.5	33	91	9.1
3	43	91.7	8.2	40	78	18.3	34	89.8	9.2	34	92.2	8.7
4	104	90.3	8.9	94	82	15.9	90	90.5	10.5	79	91.3	9.7
5 (non-urgent)	8	95.2	4.3	8	82	11.8	6	95	4.8	8	88.1	14.3
Income Quintile		*p* = 0.12			*p* = 0.16			*p* = 0.44			*p* = 0.27	
1 (lowest income quintile)	25	90.2	9.5	21	73.9	21.1	18	89	9.9	13	93	9.3
2	25	88.3	11.7	23	74.8	18.6	20	87.4	10.2	18	88.2	10.6
3	43	93	6.9	38	83.9	17.6	37	92.3	9.6	38	92.6	10.9
4	39	88.6	9.4	35	75.6	17.5	31	90.8	10.8	33	89.1	10.8
5 (highest income quintile)	72	91.4	8.1	68	77.8	16.5	63	90.2	8.9	59	92.2	7.6
Injury		*p* = 0.89			***p*** ** < 0.001**			*p* = 0.99			*p* = 0.95	
Head injury	18	91.4	6.7	17	89.3	8.2	15	91.4	6.4	12	93.6	5.9
Lower extremity fracture	25	92.9	9.2	22	58.6	19.9	16	88.7	9.4	15	90.2	10.2
Major trauma	16	89.0	11.6	15	65.5	21.2	13	90.0	9.8	13	90.0	11.2
Minor external injury	77	90.8	9.4	67	84.8	15.3	60	89.2	11.6	57	91.3	10.9
Upper extremity fracture	49	89.6	8.5	46	77.9	12.2	41	91.5	8.8	41	92.6	7.3
Other^*^	16	90.7	6.9	15	74.8	16.9	11	90.6	5.9	10	92.0	9.1

^a^Variable ranges from 0-100 with 100 representing perfect health; minimal clinically important difference is a 4 point change

*P values for differences in mean PedsQL scores across variables (within time points) from bivariable linear regression, < 0.001 considered significant with Bonferroni correction

At 12 months post injury the prevalence of outstanding impact on total HRQoL was 8 %, 10 % and 9 % for the total summary score, physical and psychosocial domains respectively (Table 4). No injury or demographic variables were associated with the prevalence of outstanding HRQoL impact overall at 12 months.

**Table 4:** Prevalence of impaired HRQoL (as defined > 1 standard deviation of baseline mean below baseline score) by injury severity

	One Month			Four Months			Twelve Months		
	Total	Physical	Psycho-social	Total	Physical	Psycho-social	Total	Physical	Psycho-social
Full population n (%)									
	90 (44.1)	113 (55.4)	58 (28.4)	23 (11.3)	28 (13.7)	18 (8.8)	13 (6.4)	16 (7.8)	15 (7.4)
Hospitalization Status n (%)									
	***p*** ** < 0.001**	***p*** ** = 0.001**	***p*** ** < 0.001**	*p* = 0.91	*p* = 0.03	*p* = 0.21	*p* = 0.87	*p* = 0.05	*p* = 0.83
ED	49 (34.0)	69 (47.9)	30 (20.8)	16 (11.1)	15 (10.4)	15 (10.4)	11 (7.6)	11 (7.6)	13 (9.0)
Hospitalized	41 (68.3)	44 (73.3)	28 (46.7)	7 (11.7)	13 (21.7)	3 (5.0)	5 (8.3)	10 (16.7)	6 (10.0)
PaedCTAS n (%)									
	*p* = 0.06	*p* = 0.18	*p* = 0.03	*p* = 0.84	*p* = 0.14	*p* = 0.85	*p* = 0.12	*p* = 0.11	*p* = 0.04
1 (resuscitation required)	7 (63.6)	7 (63.6)	6 (54.5)	1 (9.1)	2 (18.2)	1 (9.1)	0 (0.0)	0 (0.0)	1 (9.1)
2	21 (55.3)	26 (68.4)	15 (39.5)	6 (15.8)	9 (23.7)	2 (5.3)	5 (13.2)	7 (18.4)	5 (13.2)
3	21 (48.8)	24 (55.8)	13 (30.2)	5 (11.6)	7 (16.3)	4 (9.3)	1 (2.3)	2 (4.7)	1 (2.3)
4	36 (34.6)	50 (48.1)	21 (20.2)	10 (9.6)	9 (8.7)	10 (9.6)	8 (7.7)	10 (9.6)	9 (8.7)
5 (non-urgent)	5 (62.5)	6 (75.0)	3 (37.5)	1 (12.5)	1 (12.5)	1 (12.5)	2 (25.0)	2 (25.0)	3 (37.5)

*p values comparing prevalence of impaired HRQoL across groups from chi square test or Fisher’s where cell size < 5; < 0.001 considered significant with Bonferroni correction

**Fig. 2:**
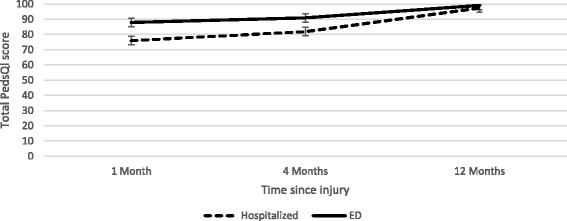
GEE estimates for injuries requiring hospitalization vs ED visit*. *Holding all other variables in model constant as: female; PaedCTAS of 1 or 2; 7.1 years of age (median age of population) and baseline PedsQl of 90.7 (mean of population)

Table 5 presents the results of the GEE model examining predictors of HRQoL over time (from 1-12 months) the QICu of the model with only time was 98 146 and the model with all covariates the QICu was 72 322. The model demonstrates that the only significant modifiers of HRQoL recovery following injury, after controlling for baseline HRQoL, were age and hospitalization status. Children who were hospitalized had a steeper slope to recovery as demonstrated by the fact that despite having lower HRQoL at one month post injury relative to children who were not hospitalized, HRQoL for both hospitalized and un-hospitalized children returned to baseline by four months post injury (Fig. 2). The parameter estimate for the adjusted model from Table 5 for the hospitalization and time interaction term can be interpreted as follows: during the time from 1-12 months post injury, in a one month period the average change in HRQoL score for children who were not hospitalized was 0.93 points less than children who were hospitalized controlling for baseline HRQoL, age, sex and PaedCTAS. Likewise, relative to children who were one year younger, older children experienced a 0.07 point greater increase in their HRQoL score in a month period, or the slope of HRQoL over time for children who were one year older was found to be 0.07 steeper than that of younger children.

**Table 5:** PedsQl Total score at 1, 4 and 12 months using Generalized Estimating Equation

	Time only Model (95 % CI)	Crude Estimate^a^ (95 % CI)	Adjusted Estimate^b^ (95 % CI)
Intercept	81.47 (79.37, 83.56)		34.53 (17.88, 51.17)
Time in months	0.99 (0.79, 1.19)		0.54 (–1.37, 2.46)
Baseline HRQoL		0.52 (0.24, 0.79)	0.53 (0.29, 0.77)
Time*Baseline		0.01 (–0.01, 0.03)	0.01 (–0.01, 0.03)
Hospitalization Status		13.65 (8.38, 18.93)	11.95 (5.59, 18.30)
Time*Hospitalization (ref = hosp)		–1.17 (–1.67, –0.68)	–0.93 (–1.58, –0.29)
Age (yrs)		–1.03 (–1.46, –0.59)	–0.93 (–1.32, –0.54)
Time*Age		0.07 (0.03, 0.12)	0.07 (0.04, 0.11)
Sex (ref = Female)		–0.41 (–5.06, 4.25)	2.63 (–1.25, 6.5)
Time*Sex		–0.10 (–0.53, 0.33)	–0.29 (–0.67, 0.10)
PaedCTAS 1&2		Reference	
PaedCTAS 3		7.51 (0.54, 14.49)	–0.59 (–8.33, 7.16)
PaedCTAS 4&5		11.87 (6.24, 17.51)	2.29 (–4.56, 9.16)
Time*CTAS3		–0.61 (–1.25, 0.02)	–0.02 (–0.73, 0.69)
Time*PaedCTAS 4&5		–1.07 (–1.58, –056)	–0.36 (–1.03, 0.32)

^a^“Crude” models include the covariate, time and the interaction between time and the covariate

^b^Adjusted for all other variables in table including interaction terms

Although injury severity, as measured by hospitalization, increased the rate of recovery, it did not impact the state of recovery (a child’s HRQoL score at a given timepoint) beyond one month post injury. The mean HRQoL score for children who were hospitalized was significantly lower than those who presented to ED at one month post injury (65.9 vs 82.6, *p* <0.001) however this difference was no longer evident at four or 12 months (Table 3). Likewise children with lower PaedCTAS scores had significantly lower HRQoL scores one month post injury, a difference that again disappeared by four and 12 months. When broken down into psychosocial and physical components of HRQoL however, we found that a greater proportion of children who were hospitalized continued to have diminished scores for the physical component of the HRQoL measurement through to 12 months post injury (16.7 % of hospitalized children vs 7.6 % of children presenting to ED, *p* = 0.05) although there was not a significant difference in the psychosocial domain of HRQoL at four or 12 months between these two groups (Table 4).

## Discussion

In accordance with previous work [13, 25], we found that most children’s summary HRQoL score had returned to within one standard deviation of baseline by four months post injury. Hospitalization status and age were the only variables associated with a significant change in the rate of recovery, with children who were admitted and older children having a faster rate (steeper slope) to recovery relative to those who were seen in the ED and younger children. This finding demonstrates that children with greater impact on HRQoL at one month post injury (those hospitalized and older children) recover at an accelerated rate and by four months post injury there is no difference in HRQoL impact relative to their ED and younger peers. Hospitalized children may have experienced a greater impact on HRQoL at one month relative to children seen in the ED due to time away from school/peers, and injuries that resulted in a greater impact on activities of daily living. Older children (those > 5 years of age) may have experienced a greater impact on HRQoL at one month post injury as they are more independent relative to younger children in activities of daily living and leisure activities. Thus, their injuries may have resulted in a greater loss of independence.

Our findings are consistent with the findings of the UK burden of injury multicenter study that recruited almost 300 hundred participants under 18 years of age. They found that admission status and injury severity were the only variables associated with recovery at one month post injury among 5-17 year olds, and that 91 % of participants had recovered by 12 months post injury [25]. In addition, Polinder et al., reported that less than 10 % of their study population of injured children 5-14 years had residual disability after nine months with girls and hospitalized children having higher odds of longer lasting disability [13]. Further, a 2012 systematic review on studies of children who have suffered traumatic brain injuries found that the odds of experiencing poor Quality of Life increased with more severe injuries (assessment time points ranged from three months to five years) [26]. However, even among children with TBI, a recent study found that by 18 months post injury parent ratings of children’s HRQoL returned to the normal range for most children, regardless of injury severity [27].

In the current study, at four and 12 months a higher proportion of hospitalized children, relative to their unadmitted counterparts, still had physical HRQoL scores that were at least one standard deviation lower than their baseline score while their total and psychosocial scores were on par with baseline. PaedsCTAS did not have a significant impact on recovery despite being a measure of severity. It is possible this result is due to the fact that although PaedsCTAS has been found to be associated with physical recovery, no relationship with psychosocial recovery has been observed [19]. Future analysis will explore predictors of physical and psychosocial functioning independently. The utility of providing targeted rehabilitation support, such as occupational and physical therapy, throughout recovery to injured children who were hospitalized to help diminish this impact could be investigated in future research.

Variables that could be used to predict and protect the subset of children at high risk of long-term or more serious impact, outside of lengthy hospitalization and possibly severe traumatic brain injury [28, 29] have not been consistently demonstrated across studies. Some studies have found that children involved in motor vehicle accidents [30] and burn victims [31] can have more psychological and/or longer lasting sequalae relative to other injuries; the sample size of children with these mechanisms of injury in the current study was too small to investigate this further.

Our findings can inform the debate regarding the tradeoff between the benefits of a physically active lifestyle versus potential impacts on HRQoL resulting from childhood injuries [32–38]. Among our sample, 63 % were engaged in leisure/physical activity at the time of their injury, highlighting the high incidence of these injuries. Our data did not include information on exposure time, however, a systematic review calculated that the injury incidence rate during leisure/physical activity was between 0.15-0.27 medically attended injuries per 1,000 h of physical activity, indicating that while they may be prevalent, they are relatively rare when accounting for exposure [39]. In those relatively rare cases when injury does occur, our findings suggest that most children recuperate quickly, with HRQoL comparable to pre-injury levels by four-months post-injury.

The limitations of this study should be noted in interpreting the findings. Despite our best efforts, the study sample represents 30 % of the eligible population that was approached for study participation. Our response rates appear to be lower than other comparable longitudinal injury studies of children attending an ED or admitted to hospital for an injury. For example, Polinder et al.’s pediatric study had a response rate of 43 % [13], while Lyons et al.’s study of injured children and adults had a participation rate of 66 % [25, 40]. However, when we compared the study population to the broader population of children presenting at BCCH with an injury, we found that our sample matched all injured children, with the exception of income with our study population having a significantly lower proportion of individuals from the lowest two income quintiles. It is possible that being from a lower income bracket could be associated with a detrimental impact on HRQoL recovery, which may not have been captured in this study due to small sample size. The influence of income was not examined in Polinder et al. and Lyons et al.’s research, thus limiting our understanding of this issue. Our sensitivity analysis indicated no significant differences in HRQoL over time resulting from excluding participants due to missing data.

We were successful in sampling a breadth of injuries and over-sampling injuries that required hospitalization. As with any longitudinal research, there was attrition over the course of this study. Those lost to follow-up had a lower mean baseline HRQoL score relative to those who completed the study period; however, this difference was less than the minimal clinically important difference of 4.5 points [41]. Finally, baseline health prior to injury was based on a retrospective measure and it is possible that parents under- or over-represented child health prior to injury. It has been suggested that baseline measures collected at recruitment are more appropriate than healthy population norms for the purpose of determining the impact of injury on HRQoL in an adult population [42]. No study, to our knowledge, has examined this in a pediatric population.

## Conclusions

This study examined the longitudinal recovery of children in the year following injury. Our findings indicate that very few injuries have a long lasting impact on children’s HRQoL, demonstrating children’s resilience to physical trauma. This research contributes to, and expands upon the current literature on recovery from childhood injury by including a wide age-range of children, looking at a longer time period post-injury, and using a pediatric tool to measure HRQoL. Older and hospitalized children experienced greater short-term impact to HRQoL and a steeper slope to recovery. On average, hospitalized children continued to experience greater impact to the physical domain of their HRQoL throughout the year post-injury. On-going rehabilitation support should be considered as a mechanism to reduce physical sequalae. Overall, the rapid recovery trajectory for most injuries encourages children’s participation in active healthy lifestyles.

## Abbreviations

BC: British Columbia
BCCH: British Columbia children’s hospital
ED: Emergency department
GEE: Generalized estimating equation
HRQoL: Health related quality of life
PaedCTAS: Pediatric Canadian triage and acuity scale
PedsQL™: Pediatric quality of life questionnaire
